# Presence of cancer-associated mutations in exhaled breath condensates of healthy individuals by next generation sequencing

**DOI:** 10.18632/oncotarget.15233

**Published:** 2017-02-09

**Authors:** Omar Youssef, Aija Knuuttila, Päivi Piirilä, Tom Böhling, Virinder Sarhadi, Sakari Knuutila

**Affiliations:** ^1^ Faculty of Medicine, Department of Pathology, University of Helsinki, Helsinki, Finland; ^2^ Department of Pulmonary Medicine, University of Helsinki and Helsinki University Hospital, Heart and Lung Center, Helsinki, Finland; ^3^ Unit of Clinical Physiology, HUS-Medical Imaging Center, Helsinki University Hospital and Helsinki University, Helsinki, Finland; ^4^ Department of Pathology, University of Helsinki and HUSLAB, Helsinki University Hospital, Helsinki, Finland

**Keywords:** exhaled breath condensate, mutations, healthy individuals, next generation sequencing

## Abstract

Exhaled breath condensate (EBC) is a non-invasive source that can be used for studying different genetic alterations occurring in lung tissue. However, the low yield of DNA available from EBC has hampered the more detailed mutation analysis by conventional methods. We applied the more sensitive amplicon-based next generation sequencing (NGS) to identify cancer related mutations in DNA isolated from EBC. In order to apply any method for the purpose of mutation screening in cancer patients, it is important to clarify the incidence of these mutations in healthy individuals. Therefore, we studied mutations in hotspot regions of 22 cancer genes of 20 healthy, mainly non-smoker individuals, using AmpliSeq colon and lung cancer panel and sequenced on Ion PGM.

In 15 individuals, we detected 35 missense mutations in *TP53, KRAS, NRAS, SMAD4, MET, CTNNB1, PTEN, BRAF, DDR2, EGFR, PIK3CA, NOTCH1, FBXW7, FGFR3, and ERBB2*: these have been earlier reported in different tumor tissues. Additionally, 106 novel mutations not reported previously were also detected. One healthy non-smoker subject had a KRAS G12D mutation in EBC DNA.

Our results demonstrate that DNA from EBC of healthy subjects can reveal mutations that could represent very early neoplastic changes or alternatively a normal process of apoptosis eliminating damaged cells with mutations or altered genetic material. Further assessment is needed to determine if NGS analysis of EBC could be a screening method for high risk individuals such as smokers, where it could be applied in the early diagnosis of lung cancer and monitoring treatment efficacy.

## INTRODUCTION

In spite of recent improvements in the treatment of many cancers, the prognosis of lung cancer has remained unchanged for 20 years and lung cancer is still the leading global cause of cancer related deaths [1]. This is mainly due to the lack of early screening and suitable diagnostic markers, resulting in diagnosis of the disease only at a late stage. When a tumor is at an advanced stage, molecular pathogenesis has progressed to a level where there are numerous genetic and epigenetic changes, allowing cancer cells to be naturally resistant or to rapidly develop resistance to treatments, even to the new targeted tyrosine kinase inhibitors such as that for EGFR [2].

Developments in microarray based techniques, next generation sequencing (NGS) and bioinformatics tools have made it possible to identify genome-wide gene alterations from an extremely small amount (1-10 ng) of DNA or RNA [3–5]. In turn, this has made it possible to use exhaled breath condensate (EBC) as a source of testing material since this is a patient-friendly, non-invasive approach. We are one of the first groups to successfully use the NGS approach for EBC analysis, as illustrated in our recent review article [6]. Genetic changes in EBC DNA are thought to reflect alterations present in lung tissue and the sampling process is convenient for the patient and the specimens can be collected repeatedly throughout the follow-up [6].

Numerous recurrent somatic mutations have been well characterized in lung cancer and their predictive value and prognostic significance are widely acknowledged [7–9]. However, very little, if anything, is known about the presence of these mutations in cells or cell-free DNA of non-malignant, seemingly healthy individuals. As EBC may open a promising route for early diagnosis and follow up of lung cancer, it is extremely important to determine whether mutations thought to be tumor-associated may also be present in healthy subjects. This kind of basic information is needed before any firm conclusion can be drawn on the significance of mutations in EBC of lung cancer patients. In this study, we describe the presence of hot spot mutations in healthy subjects, even in non-smoking individuals.

## RESULTS

The sample volume collected after 15 minutes of breathing ranged in size from 1.5 ml to 4.0 ml (mean 3.1 ml). The average DNA yield obtained from the EBC specimens (1.5-4.0 ml) was 75.5ng. NGS was successful in all but one subject (success rate 95.5%). The average mean depth was 901 while the average percentage of reads on target was 83.7%. All sequencing data are shown in Supplementary Table 1.

Two subjects (9.5%) did not display any evidence of mutations in their specimens, three others (14.4%) showed only novel but no hotspot mutations, while the remaining fifteen (76.1%) exhibited various types of genetic mutations.

### Hotspot mutations

**Figure 1:**
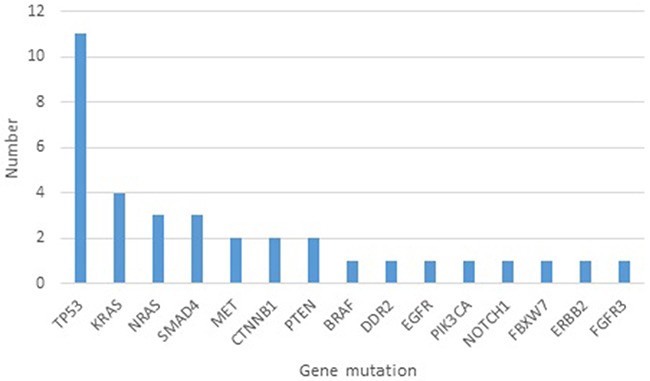
Number of hotspot mutations in different genes detected in exhaled breath condensates of 20 healthy subjects

Hotspot mutations refer to those somatic mutations that have been reported earlier and are recorded in COSMIC database. The number of hotspot mutations in the different genes is shown in Figure 1. In all, 35 hotspot mutations were detected in the EBC from our 20 healthy individuals. *TP53* was the gene most frequently mutated with 11 mutations detected in eight subjects (40%) (five females and three males). Three individuals had two mutations in the *TP53* at different positions, while the remaining five subjects showed only one mutation. *TP53* mutations were the most frequent ones concurrently occurring with mutations in other genes such as *PTEN, MET, EGFR, SMAD4, CTNNB1, BRAF*, and *KRAS*.

*KRAS* mutations were the second most frequently encountered (seen in three subjects: one male and two females). Importantly, one subject carried codon 12 mutation (G12V). *SMAD4* mutations were found in three individuals (14.4%), and *NRAS* mutations were detected in two individuals (9.5%) with one subject harboring two different *NRAS* mutations, while two other subjects displayed *CTNNB1* gene mutations.

### Novel variants

**Figure 2:**
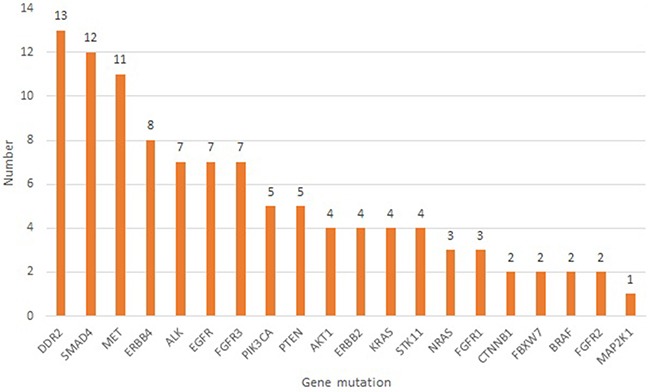
Number of all novel non-synonymous mutations detected in exhaled breath condensates of 20 healthy subjects

A total of 106 novel mutations were found that led to an amino acid change (including missense, nonsense and indels) and which had not been reported previously in either the COSMIC or dbSNP databases. The most frequent novel mutations were in *DDR2, SMAD4, MET* followed by *ERBB4, ALK, EGFR, FGFR3* then *PIK3CA, PTEN, AKT1, ERBB2, KRAS, STK11, NRAS, FGFR1, CTNNB1, FBXW7, BRAF, FGFR2*, and *MAP2K1* while no novel mutations reported for *TP53* as shown in Figure 2. All COSMIC hotspots and non-synonymous novel mutations seen in each sample are shown in Table 1.

**Table 1:** Sequencing results and description of healthy subjects included in the exhaled breath condensate (EBC) analysis

EBC sample	Age	Gender	Smoking status	Hotspot mutation					Novel mutation		
				Gene	Position	Variant allele fraction (%)	Previously reported in tissue (COSMIC)	Number of mutations reported (COSMIC)	Gene	Position	Mutant allele fraction (%)
1	31	M	Never	*KRAS*	p.G12V	6.8	Lung & various	1412 in lung & 9091 total	*ALK*	p.G1263R	8.4
									*ALK*	p.P1153S	9.4
									*ERBB2*	p.K854R	5.5
									*ERBB4*	p.R306H	5.4
									*KRAS*	p.D126N	3.5
2a*	68	M	Never						*BRAF*	p.R603L	9.8
									*BRAF*	p.E586*	5.6
									*FGFR1*	p.A290V	5.6
									*NRAS*	p.S17G	12
2b*	68	M	Never	*FGFR3*	p.H274Y	7.3	Various	1	*DDR2*	p.F242S	27.8
				*CTNNB1*	p.T41A	4.3	Various	979	*EGFR*	p.N476D	4.6
									*FGFR3*	p.S802R	9.4
									*STK11*	p.D352E	10.2
									*PTEN*	p.D326N	5.5
3	49	F	Never	*KRAS*	p.D30Y	4.7	Various	1	*DDR2*	p.E779G	9.9
				*KRAS*	p.T20M	3.9	Various	3	*EGFR*	p.L704S	4.5
				*TP53*	p.G112S	26.5	Various	2	*EGFR*	p.L760F	8.7
				*TP53*	p.I251T	3.6	Various	3	*ERBB4*	p.F297L	7.6
									*ERBB4*	p.M343V	3.9
									*FGFR3*	p.K710E	4.1
									*SMAD4*	p.T338A	6.3
									*PIK3CA*	p.A1020T	3.8
									*PTEN*	p.K267R	11.5
4	56	F	Never	*NRAS*	p.D54G	4.8	Various	1	*DDR2*	p.G104W	15.9
				*NRAS*	p.A11T	5.4	Various	3	*DDR2*	p.G108V	88.5
									*DDR2*	p.F593C	5.8
									*DDR2*	p.S598G	10.7
									*DDR2*	p.E786D	4.6
									*EGFR*	p.N493S	5.5
									*SMAD4*	p.H444R	11.9
									*ERBB4*	p.F583L	5.6
									*STK11*	p.P203Q	22.7
5	26	F	Never	*TP53*	p.P72R	4.6	Pleura. upper aerodigestive & various	1 in pleura & 38 total	*ALK*	p.C1182Y	51.7
									*PIK3CA*	p.A1027T	13.1
									*ERBB2*	p.G865R	33
6	51	F	ex-smoker	*PIK3CA*	p.M1043V	17.8	Lung & various	1 in lung & 38 total	*FGFR3*	p.Q256R	6.5
									*SMAD4*	p.V409A	5.4
									*DDR2*	p.P116L	5.6
									*CTNNB1*	p.M12V	99
7	48	F	Never	*MET*	p.R359L	6.3	Various	1	*MET*	p.V177G	4.1
				*PTEN*	p.F258L	9.2	Various	1	*MET*	p.G344E	9.7
				*TP53*	p.S166P	11.2	Various	5	*MET*	p.D372N	17.4
									*MET*	p.C1109Y	5.7
									*MET*	p.V1255A	5.6
									*MET*	p.D358N	9.8
									*MET*	p.H1256Q	9.1
									*KRAS*	p.D54Y	5.6
									*SMAD4*	p.T197A	14.2
8	25	M	Never	*…*	…	…	…	…	*…*	…	…
9	38	F	Never	*EGFR*	p.D761N	11.5	Lung & various	2 in lung & 5 total	*ALK*	p.N1175D	11
				*SMAD4*	p.S171L	3.7	Various	1	*FGFR1*	p.R285Q	12.9
				*TP53*	p.Q104*	20.3	Lung & various	4 in lung & 24 total	*PTEN*	p.L181Q	4.7
									*AKT1*	p.Y26C	5.3
									*SMAD4*	p.Y117C	4.5
									*PIK3CA*	p.C695R	9.6
									*EGFR*	p.I490V	6.7
									*ERBB2*	p.S792P	4
									*STK11*	p.A206V	4.7
10	27	F	Never	*…*	…	…	…	…	*DDR2*	p.C784R	4.4
									*ALK*	p.I1194F	3.8
									*EGFR*	p.P794L	7.3
									*KRAS*	p.T20A	4.1
									*FGFR3*	p.S402I	4.3
									*SMAD4*	p.N188S	4.7
11	28	F	ex-smoker	*TP53*	p.Y163*	11.0	upper aerodigestive & various	5	*ERBB4*	p.D245G	6
				*TP53*	p.F134L	5.5	Various	29	*FGFR1*	p.N180S	7.9
12	21	M	ex-smoker	*KRAS*	p.A11V	5.5	Lung & various	1 in lung & 3 total	*EGFR*	p.Y764D	14.2
				*SMAD4*	p.F408S	9.6	Various	1	*PTEN*	p.V249A	4.5
									*KRAS*	p.L53F	16
									*DDR2*	p.H110R	8.4
									*CTNNB1*	p.D17G	10
									*MET*	p.F374L	6.6
									*SMAD4*	p.N468S	4.5
13	27	M	Never	*CTNNB1*	p.D32Y	10.6	Various	187	*NRAS*	p.L52S	8.3
									*ERBB4*	p.V590A	5.1
									*FGFR3*	p.F706S	4.7
									*FGFR3*	p.S783I	21.7
									*SMAD4*	p.V397A	8
14	25	F	Never	*…*	…	…	…	…	*FBXW7*	p.T570A	9.1
15	31	M	Never	*SMAD4*	p.A452T	56.1	Various	1	*DDR2*	p.A107V	6.1
				*NOTCH1*	p.V1578delV	4.2	Various	20	*ALK*	p.L1187P	9.5
				*NRAS*	p.G60E	25.2	Various	7	*PIK3CA*	p.L540P	6.2
				*PTEN*	p.L57S	100	Various	2	*MET*	p.V1110A	14.1
									*ERBB2*	p.F864S	6.3
									*MET*	p.V1007I	12.9
16	33	M	Never	*TP53*	p.R337C	26.6	Various	41	*NRAS*	p.F28S	23.7
				*TP53*	p.N345D	24.2	Various	1	*DDR2*	p.D661N	11.9
									*ALK*	p.D1203G	4.3
									*ERBB4*	p.N269D	3.9
									*PIK3CA*	p.N1068S	3.4
									*FBXW7*	p.I435V	4
17	34	M	Never	*MET*	p.S349G	6.5	Various	1	*FGFR2*	p.L390S	9.8
				*DDR2*	p.R105C	16.7	Various	1	*AKT1*	p.R25H	6.8
				*TP53*	p.M169I	9.2	Lung & various	1 in lung & 9 total	*DDR2*	p.H246Y	8
									*PTEN*	p.K237E	11.2
									*SMAD4*	p.P185L	9.5
									*STK11*	p.D355G	4.1
18	46	F	ex-smoker	*BRAF*	p.K601E	12.1	Lung & various	3 in lung & 146 total	*FGFR2*	p.C555Y	6.4
				*ERBB2*	p.V773M	49.0	Various	1	*MAP2K1*	p.L50H	9
				*FBXW7*	p.R278*	19.2	Various	24	*SMAD4*	p.H184R	14.3
									*SMAD4*	p.P470S	5.1
									*SMAD4*	p.N316S	42.1
									*MET*	p.S187G	4.6
									*ERBB4*	p.A345T	29
									*FGFR3*	p.G372S	6.5
19	38	M	Never	*…*	…	…	…	…	*…*	…	…
20	32	M	Current smoker	*TP53*	p.S269N	7.0	Various	5	*AKT1*	p.F27V	8.1
									*AKT1*	p.L28F	4.3

* Two different specimens from the same individual with one month interval.

## DISCUSSION

As far as we are aware, this is the first study to use NGS to analyze mutations in EBC of healthy individuals. A total of 35 hotspot mutations and 106 novel mutations were detected. The genes with the most frequent hotspot mutations in order from top to bottom were: *TP53, KRAS, NRAS, SMAD4, MET, CTNNB1, PTEN*, *BRAF, DDR2, EGFR, PIK3CA, NOTCH1, FBXW7, FGFR3*, and *ERBB2*.

In the present study, 11 different *TP53* mutations were seen, of which only three (Q104*, Y163*, and M169I) have been reported previously in lung tissues and upper aerodigestive tract according to COSMIC database. In addition, another *TP53* mutation (P72R) has been reported in pleural tissue from a mesothelioma patient. The remaining eight *TP53* somatic mutations have been reported in the COSMIC database in other types of malignancies such as colon cancer, breast cancer and hematological malignancies. There is one similar finding of *TP53* mutations in the cell-free circulating DNA in 11% out of 205 non-cancerous control subjects, and in 35.7% early-stage and 54.1% late-stage small cell lung carcinoma (SCLC) patients [10]. A prospective study demonstrated the presence of both *TP53* (3.2%) and *KRAS* (1%) mutations in the plasma of healthy individuals. The authors reported that the patients remained clinically cancer-free after five years of follow up [11]. Another approach, exploiting an ultra-deep sequencing technique, was able to detect a low frequency of *TP53* mutations in peritoneal fluid of all non-cancerous controls [12].

Four *KRAS* hotspot mutations were seen in three individuals with one subject harboring clinically important codon 12 mutation. The previous study by Kordiak et al, using mutant-enriched PCR technique on EBC specimens [13], detected codon 12 *KRAS* mutations in 26 normal individuals (out of 52 control subjects) and in 11 patients with benign pulmonary lesions. Moreover, they detected mutated *KRAS* in the normal pulmonary tissue parenchyma excised from patiensts with lung cancer. The authors considered that this was attributable to the release of DNA from pulmonary cells through apoptosis, necrosis or spontaneous active release processes into airway epithelial lining fluid and thus into EBC. Similarly, two other studies were able to detect *KRAS* mutations in the sputum of 12.5% normal individuals compared to the 48% detection rate in cancer patients. The mutations could be detected only by applying highly sensitive enriched PCR, indicating that only a few cells carried this mutation [14, 15].

By using Ion Torrent NGS technology, *KRAS* mutations have been reported in plasma of 3.7% of healthy controls and 4.3% of patients with chronic pancreatitis [16]. These investigators noted that the mutant allele fraction was significantly lower (0.2% to 1%) when compared to the mutant *KRAS* allele fraction in patients with pancreatic cancer (1% to 50%). The authors speculated that somatic mutations occur at negligible frequencies in the normal cell population. Similarly, another study reported the finding of *KRAS* mutations in tissue specimens from patients with colitis, hyperplastic polyps, and normal colonic mucosa that did not have any kind of neoplasia [17].

**Figure 3:**
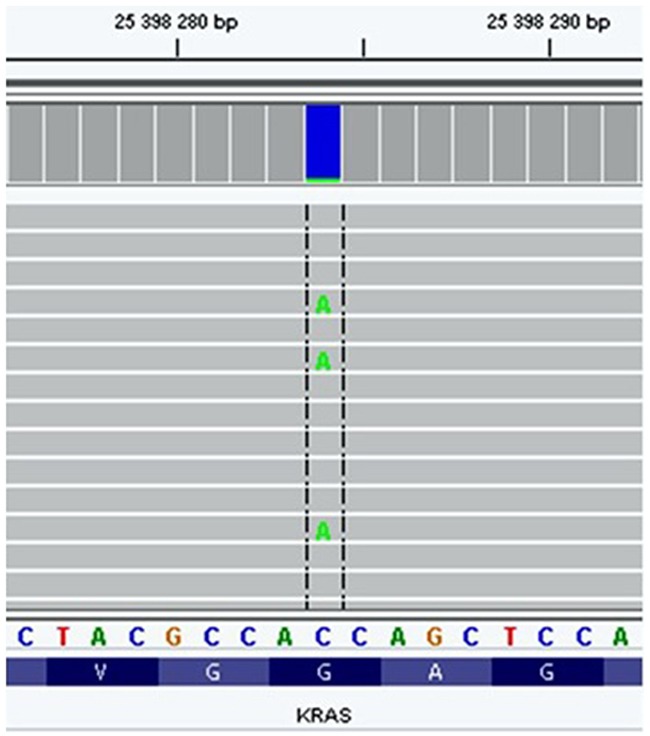
KRAS: G12V mutation detected in the EBC sample from a healthy nonsmoking subject

In our study, one specimen exhibited the clinically relevant codon 12 *KRAS* mutation (G12V) with a mutant allele fraction of 6.8% (Figure 3). This codon mutation was found to be the most frequent mutation in tumor tissue in our previous study of Finnish NSCLC and has also been often described in tissues of lung cancer in other studies [18]. This is in concordance with a recently published study that reported the detection of *KRAS* G12V mutation in the plasma of three out of six controls, at low concentration (1.25 to 1.87 copies/mL) by using droplet digital PCR [19].

*NRAS* mutations were detected in two of our subjects with one subject harboring 2 different mutations. The other subject had an *NRAS* mutation in association with other hotspot alterations; *NOTCH1, PTEN* and *SMAD4*.

The *EGFR* mutation (D761N) was seen in one of our EBC samples from a female never-smoker, while *BRAF* (K601E) was present in a normal ex-smoker subject. Our small sample size, and only one current smoker does not allow to analyze mutations in relation to the smoking status. Two mutations, p.D32Y and p.T41A, in the beta-catenin gene (*CTNNB1*) found in our healthy subjects have been reported in tumors of large intestine, hepatic and endometrial cancers, according to the COSMIC database. Additionally, a *NOTCH1* mutation (p.V1578delV) was found in one EBC sample. This mutation is frequently seen in cancers of lymphoid origin but it has been reported also in non-malignant periprosthetic soft tissue masses (pseudotumors) from patients with metal on metal hip replacement [20]. Two *MET* mutations in two individuals occurred along with *TP53* mutations, and *SMAD4* mutations were seen in three individuals.

From one of our subject (EBC 2), we sampled EBC twice, with a gap of one month to compare the sequencing results. Although the sequencing depth from one of the replicates was not very good, most of the germline SNP (except those where the amplicon did not amplify in one sample with inadequate sequencing libraries) were detected in both of the samples. The somatic mutations were however not common in the repeated sample.

Thus, our results clearly demonstrate the presence of hotspot mutations in EBC from healthy individuals. In interpretation of positivity, we set our threshold for mutant allele frequency to a minimum of 3% based on our previous comparison of *EGFR* and *KRAS* mutations as detected by NGS and clinically approved PCR methods from FFPE samples [21]. Of the total 35 hotspot mutations detected in our healthy subjects, there were 26 mutations that had a mutant fraction of 5% or more, of which 16 had more than 10%. Importantly, the clinically relevant KRAS codon 12 mutation seen in one subject had a mutant fraction of 6.8% (coverage, 1398). From a methodological point of view, before any firm conclusions can be drawn regarding the clinical significance of these mutations, it will be necessary to conduct a comparison of the mutant allele fraction in larger series of lung cancer patients and normal healthy individuals. A prospective study has reported an association between the presence of the codon 12 *KRAS* mutation in plasma of apparently healthy individuals and the development of bladder cancer after a follow-up period [11]. Therefore, RAS pathway activation may cause early changes that could contribute to tumor development [22]. However, the significance of these hotspot mutations in normal subjects needs to be clarified. The highly sensitive NGS technique used in this study could partially explain the detection of these hotspot mutations in healthy individuals. The presence of mutations, despite the relatively younger age of the normal subjects in our study compared to lung cancer patients, could indicate that they may be a part of an apoptotic process occurring in normal lung parenchyma. It might thus reflect the mutagenic load that normal cells are exposed to as a result of environmental factors such as air pollution, asbestos exposure, active and/or passive smoking [23]. For tissues to maintain cellular homeostasis, cells with unrepaired DNA damages are eliminated and can be detected by sensitive methods [24]. This would agree with earlier reports describing the presence of both *TP53* and *KRAS* mutations in normal subjects who did not develop a malignancy during their follow-up [11]. On the other hand, they may reflect molecular changes occurring in lung tissues of healthy subjects that might represent very early markers of an ongoing carcinogenesis process. Indeed, these findings might serve as indicators for disease, but their applications in clinical diagnostic procedures will require more investigations.

To conclude, in the current study, hotspot mutations were detected in EBC of 75% of healthy individuals. This could represent either a normal process of cell death and cellular renewal, or early carcinogenic changes. High throughput NGS technology now makes it possible to detect genetic mutations with high sensitivity and low allele frequencies. These observations will require further investigations to confirm whether it is possible to exploit NGS analysis on EBC DNA as a non-invasive screening method for high-risk individuals such as smokers, for example, in the early diagnosis of lung cancer. Our results highlight the importance of knowing the prevalence of cancer-related mutations, in any tissue under study, in healthy individuals before it can be applied for cancer diagnostics.

## MATERIALS AND METHODS

### EBC specimen collection procedure

EBC samples were collected from twenty adult healthy subjects with a mean age of 34.9 years. From one individual, two different specimens were taken after an interval of one month (EBC 2a and EBC 2b). Detailed information about our subjects is given in Table 1. Smoking history was noted and individuals were classified into three categories: never-smoker, ex-smoker, and current smoker. The subjects were mainly never smokers (n= 15), there was only one current smoker and four ex-smokers.

EBC was collected after 15 min of breathing into the EcoScreen instrument (Jaeger/Germany). Breathing frequency and mean breath volume were checked every 5 minutes till the end of the collection procedure. Collected EBC samples were transported on ice immediately to the laboratory. The samples were then transferred to 2ml tubes with the sample volume being measured before storage at −70°C.

The study was approved by the HUS review board (Ethical permission number 253/13/03/01/2015). Written informed consent was obtained from all subjects.

### EBC DNA extraction

DNA was extracted from the whole EBC sample (ranging from 1.5 to 4 ml) using the QIAamp circulating nucleic acid kit (Qiagen Cat NO. /ID 55114) according to the manufacturer's instructions and using a vacuum pump. Extracted DNA was eluted in 35μl of elution buffer, and then DNA was quantified by a Qubit® 2.0 Fluorometer (Life Technologies) using the Qubit® dsDNA HS Assay kit. The extracted DNA was stored at −20°C.

### Next generation sequencing of EBC

Around 10ng of DNA was used to prepare the sequencing libraries. The libraries were prepared with the Ion AmpliSeq™ Library kit 2.0 (Thermo Fisher Scientific) and with a primer pool to analyze 504 mutational hotspots and targeted regions in 22 genes commonly implicated in lung cancer: *AKT1, ALK, BRAF, CTNNB1, DDR2, EGFR, ERBB2, ERBB4, FBXW7, FGFR1, FGFR2, FGFR3, KRAS, MAP2K1, MET, NOTCH1, NRAS, PIK3CA, PTEN, SMAD4, STK11*, and *TP53*. Amplified products were purified with Agencourt AMPure XP beads (Beckman Coulter Genomics, High Wycombe, UK). Concentrations of amplified and bar-coded libraries were measured using the Qubit® 2.0 Fluorometer and the Qubit® dsDNA HS Assay kit. DNA libraries were stored at −20°C.

The libraries were clonally amplified on Ion Sphere™ particles after dilution of the libraries to 100 pM. Template preparation was performed with the Ion OneTouch™ 2 System (Thermo Fisher Scientific), an automated system for emulsion PCR, recovery of Ion Sphere™ Particles, and enrichment of template-positive particles.

The Ion Sphere™ particles coated with template were applied to the semiconductor chip. A short centrifugation step was conducted to allow the spherical particles to be deposited into the chip wells. Finally, sequencing was carried out using Ion 316™ chips on the Ion Personal Genome Machine System (PGM™, Thermo Fisher Scientific) using the Ion PGM™ Sequencing Hi-Q kit v2.

### Data analysis

The Torrent Suite Software v.4.0.2 (Life Technologies) was used to assess run performance and data analysis. Integrative Genomics Viewer (IGV v 2.2, Broad Institute) was used for visual inspection of the aligned reads. Sequencing data were further filtered and analyzed through quality checking. We selected all SNVs in the studied genes resulting in a non-synonymous amino acid change, or a premature stop codon, and all short indels resulting in either a frameshift or insertion/deletion of amino acids. All SNVs were analyzed for previously reported hotspot mutations (somatic mutations reported in COSMIC database) and novel variations, i.e. new mutations detected by NGS but not reported in either COSMIC or dbSNP databases.

## SUPPLEMENTARY MATERIALS TABLES


