# Supplementary material for: Presence of cancer-associated mutations in exhaled breath condensates of healthy individuals by next generation sequencing
Source: Oncotarget. 2017 Feb 9;8(11):18166–76. doi: 10.18632/oncotarget.15233 (PMC5392316; doi:10.18632/oncotarget.15233)
Supplement: Supplementary file 1 [file oncotarget-08-18166-s001.pdf]

Presence of cancer-associated mutations in exhaled breath condensates of healthy individuals by next generation sequencing

SUPPLEMENTARY TABLE

Supplementary Table 1: Exhaled breath condensate (EBC) volumes, DNA amounts isolated, and sequencing data details from 20 healthy individuals studied for mutations in EBC by next generation sequencing

Sample	Sample volume (ml)	Total DNA (ng)	% Reads on target	Mean depth	Uniformity
**EBC 1**	3.5	42	96.94 %	1337	97.16 %
**EBC 2a***	3.9	52.2	94.86 %	355.3	89.66 %
**EBC 2b***	3.6	60.6	89.58 %	1679	92.06 %
**EBC 3**	1.8	63.6	87.25 %	1051	90.21 %
**EBC 4**	3.5	74.1	77.56 %	437.2	69.71 %
**EBC 5**	2.8	44.8	74.03 %	173.2	78.14 %
**EBC 6**	2	110.3	84.48 %	307.9	81.09 %
**EBC 7**	2.6	104.7	90.15 %	1963	90.83 %
**EBC 8**	4	46.6	96.80 %	2153	98.31 %
**EBC 9**	2.4	74.55	82.48 %	557.4	92.79 %
**EBC 10**	3.5	39.9	90.31 %	1216	99.26 %
**EBC 11**	3.74	36.8	73.99 %	948.2	89.29 %
**EBC 12**	3.59	67.9	72.53 %	918.4	86.38 %
**EBC 13**	3.58	82.3	74.64 %	303	81.76 %
**EBC 14**	3.33	102.6	80.23 %	71.77	78.27 %
**EBC 15**	3.2	102.6	72.44 %	186.1	67.30 %
**EBC 16**	3.3	78.4	82.97 %	1451	88.01 %
**EBC 17**	2.8	117.3	79.79 %	742.9	76.82 %
**EBC 18**	3.5	124.3	79.40 %	742	72.40 %
**EBC 19**	1.74	96.3	90.08 %	1459	98.31 %
**EBC 20**	2.60	44.5	86.55 %	870.1	98.31 %
**Average**	**3.1**	**74.6**	**83.67 %**	**901.1**	**86.48 %**

*Two different specimens at one month interval
